# Microscale Thermophoresis as a Screening Tool to Predict Melanin Binding of Drugs

**DOI:** 10.3390/pharmaceutics12060554

**Published:** 2020-06-16

**Authors:** Laura Hellinen, Sina Bahrpeyma, Anna-Kaisa Rimpelä, Marja Hagström, Mika Reinisalo, Arto Urtti

**Affiliations:** 1 School of Pharmacy, Faculty of Health Sciences, University of Eastern Finland, 70210 Kuopio, Finland; laura.pelkonen@uef.fi (L.H.); sina.bahrpeyma@uef.fi (S.B.); 2 Drug Research Programme, Division of Pharmaceutical Biosciences, Faculty of Pharmacy, University of Helsinki, P.O. Box 56, FI-00014 Helsinki, Finland; anskurimpela@gmail.com (A.-K.R.); or marja.hagstrom@gmail.com (M.H.); 3 Laboratory of Biohybrid Technologies, Institute of Chemistry, St. Petersburg State University, Peterhoff, St. Petersburg 198504, Russia

**Keywords:** melanin, pigment, pigment-binding, drug delivery, eye, retina, targeting, microscale thermophoresis, screening

## Abstract

Interactions between drugs and melanin pigment may have major impacts on pharmacokinetics. Therefore, melanin binding can modify the efficacy and toxicity of medications in ophthalmic and other disease of pigmented tissues, such as melanoma. As melanin is present in many pigmented tissues in the human body, investigation of pigment binding is relevant in drug discovery and development. Conventionally, melanin binding assays have been performed using an equilibrium binding study followed by chemical analytics, such as LC/MS. This approach is laborious, relatively slow, and limited to facilities with high performance quantitation instrumentation. We present here a screening of melanin binding with label-free microscale thermophoresis (MST) that utilizes the natural autofluorescence of melanin. We determined equilibrium dissociation constants (K_d_) of 11 model compounds with melanin nanoparticles. MST categorized the compounds into extreme (chloroquine, penicillin G), high (papaverine, levofloxacin, terazosin), intermediate (timolol, nadolol, quinidine, propranolol), and low melanin binders (atropine, methotrexate, diclofenac) and displayed good correlation with binding parameter values obtained with the conventional binding study and LC/MS analytics. Further, correlation was seen between predicted melanin binding in human retinal pigment epithelium and choroid (RPE-choroid) and K_d_ values obtained with MST. This method represents a useful and fast approach for classification of compounds regarding melanin binding. Thus, the method can be utilized in various fields, including drug discovery, pharmacokinetics, and toxicology.

## 1. Introduction

Melanin is a pigment polymer that is present in various pigmented tissues in the human body. In the brain, the substantia nigra contains melanin, and it is present in many ocular tissues (including the iris, ciliary body, retinal pigment epithelium, choroid), inner ear, and skin [1]. In addition, the melanotic cells in melanoma can be densely pigmented [2]. Melanin is a macromolecule that is usually a mixture of two distinct polymers, namely black–brown eumelanin and red–yellow pheomelanin [3]. Both contain carboxylic groups and aromatic structures and they display autofluorescence [4] (for an overview of the eumelanin structure, see the abstract image). Melanin has a peak excitation wavelength at 450 nm and displays emissions from 440 nM to >800 nm [5]. Even though the autofluorescence of melanin makes immunofluorescent imaging of retina challenging, the melanin autofluorescence facilitates the use of fluorescence lifetime imaging ophthalmoscopy in ophthalmic diagnostics [5]. The autofluorescence of melanin has also been utilized in vitro to quantitate melanin in pigmented cells [6]. Furthermore, multiphoton tomography combined with fluorescence lifetime imaging enabled the monitoring of melanin distribution in human skin [7].

Several xenobiotics, including various clinically used drugs (e.g., atropine, timolol, penicillin G, chloroquine, ciprofloxacin), can bind to melanin [8,9]. In ocular tissues, melanin binding has a significant pharmacokinetic role. Accumulation of compounds in pigmented ocular tissues has been seen in humans and other primates [10], rodents [11], and rabbits [12]. Melanin binding leads to significant drug accumulation. For example, betaxolol concentration in the pigmented eye tissues in monkeys and humans was 20–150 fold compared to the neighboring non-pigmented tissues [10]. Pigment binding can also lead to alterations in drug responses. For example, atropine exerts longer mydriatic effects (dilation of the pupil) in pigmented animals compared to their albino counterparts [13,14], and pilocarpine causes longer miotic effects in pigmented rather than albino animals [15]. Melanin binding may decrease the cellular peak concentrations of free drug, but a melanin bound drug depot may also extend drug retention in the cells and prolong drug effects [11,16,17]. In fact, melanin binding may be a useful approach in targeted and prolonged drug delivery to the melanin containing tissues, such as retinal pigment epithelium (RPE) in the eye. This may be particularly beneficial in the treatment of chronic diseases, such as age-related macular degeneration [18]. On the other hand, the increased local exposure of toxic compounds might lead to adverse reactions, such as extrapyramidal effects caused by phenothiazine accumulation in the substantia nigra [19]. Therefore, melanin binding should be evaluated early during development of ocular and other pigment related therapeutics. This is an important part in the pharmacokinetic and toxicological evaluation of new chemical entities. The pigment binding field is not limited to only ocular drugs, since systemic medications may bind to the pigment in the eye and elsewhere, thereby giving rise to potential adverse effects [20]. Interestingly, the melanin targeting approach is under investigation in melanoma [21]. Other lines of investigation include melanin–metal complexes in magnetic resonance imaging [22] and melanin nanoparticles as delivery systems [23].

The first in vitro pigment-binding assays were reported more than 50 years ago [24,25,26]. The assay set-up is very simple. Isolated or synthetic melanin, or in some cases isolated pigmented organelles, namely melanosomes, are incubated with the compound of interest and after the binding equilibrium is reached, the pigment (melanin or melanosomes) is pelleted with centrifugation, and the free drug concentration is determined from the supernatant, usually with mass spectrometry. In recent, years medium-to high-throughput systems have been introduced to study melanin binding—a cassette-dosing approach was used to evaluate pigment binding of >30 compounds in a single incubation [9], and further, about 3400 compounds were studied with automated LC/MS analytics for melanin binding to generate an in silico model that correlates melanin binding with chemical structure [27]. However, this in silico model is complex and not fully transparent as structures of most compounds (internal Roche library) were not revealed. Since in silico predictions are not available for the whole research community, experimental in vitro melanin binding data is still needed to predict distribution of compounds to pigmented tissues.

In this article, we present a novel method for assessment of melanin binding. The method utilizes the autofluorescence of synthetic melanin in microscale thermophoresis (MST), and thereby avoids ligand quantitation with expensive mass spectrometry. Dissociation binding constants (K_d_) obtained by MST were compared to the binding affinities from traditional melanin binding assays. Importantly, our study suggests that K_d_ values obtained with MST can be used to predict melanin binding in the human RPE-choroid. So far, thermophoresis has mainly been applied to study binding of ligands to proteins, but it offers a robust and simple binding approach also for other affinity measurements. The method is based on different temperature induced changes in diffusion rates of ligand–target complexes, plain ligands, and target compounds [28]. Usually, the target (e.g., protein) is labeled with a fluorophore prior the binding assay, but the assay can be conducted in label-free conditions if one of the binding partners display intrinsic fluorescence [29]. The target is exposed to various ligand concentrations, the ligand–target complex is subjected to heating with infrared laser, and the mobility of these complexes is monitored with fluorescence. Ligand binding affects the diffusion rate of the complex, which can be seen by differences in the measured fluorescence. Since our MST approach utilizes the autofluorescence of melanin, it enables fast and robust melanin binding measurements with minimal consumption of compounds and melanin.

## 2. Materials and Methods

### 2.1. Preparation of Water-Soluble Melanin Nanoparticles

Synthetic melanin was purchased from Sigma Aldrich (#M8631-100 mg). Melanin nanoparticles were prepared as described earlier by Fan et al. [30], and low-binding pipette tips (Maxymum RecoveryTM pipet tips, Corning Incorporated, Salt Lake City, UT, USA) and tubes (No stick microtubes, Alpha Laboratories, Hampshire, UK) were used with both melanin and melanin nanoparticles. Briefly, melanin was dissolved into 1 N NaOH with a concentration of 2 mg/mL and stirred by vortexing. The solution was neutralized by adding an equal volume of 1 N HCl. The melanin solution was sonicated (4 × 15 s, 41% amplitude, SONICS Vibracell VCX750 Ultrasonic Cell Disrupter, Sonics & Materials, Inc., Newtown, CT, USA) and purified using centrifugal filters (Amicon^®^ Ultra-15, Ultracel^®^-30K, #UFC903024, Merck Millipore Ltd., Carrigtohill, Ireland; at 6238× *g* for 25 min (4 °C)) and three consecutive washes with sterile water (3 times 15 mL, centrifugation in the 30 K filter-tubes for 3 × 15 min at 6238× *g* (4 °C)). After the final centrifugation, the resulting nanoparticles were suspended into a small volume of sterile water and freeze-dried for 48–72 h (ModulyoD-230, Thermo Savant, Holbrook, NY, USA), and stored at −20 °C until the binding assays.

### 2.2. Melanin-Compound Incubation before Microscale Thermophoresis

Melanin binding of each of the 13 model compounds was determined (for the concentrations in the assay, see Table 1). The dilution series of drugs were prepared onto a PCR plate (4titude PCR Plate, Skirted, #4ti-0740, Brooks Life Sciences, Chelmsford, MA, USA) in phosphate buffered saline (PBS, pH 7.4). Melanin nanoparticles were suspended into PBS (pH 7.4) at 10 mg/mL and sonicated (3 × 15 s, 41% amplitude, SONICS Vibracell VCX750 Ultrasonic Cell Disrupter). The compound dilutions (10 µL) were transferred into 0.2 mL PCR tube strips (#TBS0201, Bio-Rad Laboratories, Inc., Hercules, CA, USA), and an equal volume of 1 mg/mL melanin nanoparticle dilution was added, resulting in a final melanin concentration of 0.5 mg/mL. At least three biological replicates (individually incubated dilution series) were prepared for each compound (n = 3–5). A reference sample containing only melanin nanoparticles and PBS (pH 7.4) was included in each assay to determine the unbound state in the thermophoresis experiment. Melanin nanoparticles were incubated with the compounds at room temperature for at least 2 h.

### 2.3. Microscale Thermophoresis

After >2 h incubation, the melanin–ligand complexes were mixed gently with a pipette to ensure melanin solution homogeneity and then transferred into capillaries (Monolith NT.115 Premium capillaries, #MOK025, NanoTemper Technologies GmbH, München, Germany). The samples were analyzed with a Monolith NT.115 pico device using MO.Control Software (both from NanoTemper Technologies GmbH, München, Germany) using nano-blue excitation, LED light adjusted to 60% excitation power, and infrared laser (MST power) set to high. The software was used in Expert mode to allow thermophoresis and fluorescence detection for 30 s.

### 2.4. Data Analysis

The dissociation constants (K_d_) were determined with MO.Binding Affinity Software (NanoTemper Technologies GmbH, München, Germany) using the K_d_ binding model (Equation (1) below; built-in analysis in the MO.Binding Affinity Software). The equation is based on the Langmuir binding isotherm. The optimal time region to assess binding was determined manually by selecting a region with a high signal-to-noise ratio (>5). Equation (1) is a follows:

(1) $$f\left(c\right)=Unbound+\frac{\left(Bound-Unbound\right)\times c+c\left(target\right)+K_{d}-\sqrt{{\left(c+c\left(target\right)+K_{d}\right)}^{2}-4c\times c\left(target\right)}}{2c\left(target\right)}$$

where f(c) is the fraction bound at a given ligand concentration c. Unbound is either the normalized fluorescence signal (F_norm_; MST mode) or raw fluorescence counts (initial fluorescence mode) of the target alone. Bound is either the normalized fluorescence signal or raw fluorescence counts (initial fluorescence mode) of the melanin–ligand complex. K_d_ is the dissociation constant or binding affinity, and c (target) is the final concentration of the target in the assay. The concentration of melanin nanoparticles was set to 12.5 µM, as the concentration in the assay was 0.5 mg/mL, and the estimated molecular weight for the melanin in the nanoparticles was 40 kDa, with a diameter of 4.5 nm [30].

The normalized fluorescence is the ratio of relative fluorescence of the selected region after heating (hot region) and relative initial fluorescence before the heating (cold region, i.e., relative fluorescence values before the infrared laser was switched on). The MST traces displaying the changes in the relative fluorescence over time (heating) with the selected hot and cold regions are presented in the Supplementary Materials. In the case of ligand-induced changes in the initial raw fluorescence counts, the raw fluorescence counts prior to the heating were used (initial fluorescence mode). If the ligand-induced fluorescence changes were not observed, the normalized fluorescence signals (MST mode) were used to determine the K_d_ (for additional information, see the Supplementary Materials). The 68% confidence values for K_d_ were obtained within the MO. Affinity Analysis Software.

### 2.5. Traditional Melanin Binding Assay

The traditional melanin binding assay was conducted with melanin from the porcine RPE-choroid. We used previously published methods for melanin isolation and drug binding [11]. The concentration ranges for levofloxacin, papaverine, and terazosin are presented in Table 1. Fresh melanin suspension and test compound solutions were prepared before each experiment. The melanin suspension (in PBS, pH 7.4) was sonicated in a bath sonicator for 30 min (Elmasonic S 40 H, Elma Schmidbauer GmbH, Singen, Germany) before the experiment. The incubation with test compounds took place at 37 °C on a shaker (220 rpm), and the melanin concentration in the assay was 1 mg/mL (0.15 mg/well). In the control samples, PBS buffer (pH 7.4) was used instead of the melanin suspension. Three individual replicates were made for each experiment. After 20 h incubation, the samples were centrifuged at 20,000× *g* for 20 min, and supernatant containing the unbound ligand was collected for analysis.

### 2.6. Determination of Drug Concentrations 

Samples having an estimated concentration of 0.5 µM or higher were analyzed with ultra-high pressure liquid chromatography (UPLC) (Acquity UPLC, Waters, Milford, MA, USA) with UV detection (Photodiode Array Detector, Waters, MA, USA). The separation was carried out on a Luna Omega Polar C18 (1.6 μm, 2.1 × 50 mm^2^) column (Phenomenex) at 30 °C. Injection volume was 4 µL. Gradient mode was used for all the compounds with an acetonitrile/15 mM phosphate buffer (pH 2) mobile phase. Gradient duration was 3−5 min depending on the sample (levofloxacin, terazosin, and papaverine).

Analyses of the compounds with estimated concentrations below 0.5 µM (levofloxacin, terazosin, and papaverine) were carried out with a Waters UPLC tandem mass spectrometry instrument (UPLC–MS/MS; Waters, MA, USA). All internal standards, namely levofloxacin D8, terazosin D8, and papaverine D3, were purchased from Toronto Research Chemicals (Toronto, ON, Canada). The compounds were separated with liquid chromatography on a Waters UPLC HSS T3 (1.8 μm, 2.1 × 100 mm) column at 40 °C. Injection volumes were 0.2 μL for terazosin, 0.3 µL for levofloxacin, and 0.15 µl for papaverine with a flow through needle injection system. The mobile phase consisted of 0.1% formic acid (Merck, Darmstadt, Germany) in ultrapure water (A) and 100% LC–MS grade acetonitrile (Honeywell, Seelze, Germany) (B). The gradient elution for terazosin started with 10% B at 0–0.5 min and continued with 10–95% B at 0.5–3.5 min, while the complete run time was 6.7 min, including column wash and equilibration. The gradient for papaverine started with 10–95% B at 0–3 min, while the total run time was 7.5 min. The gradient for levofloxacin was the same as for papaverine, but the total run time was 6 min. The flow rate was 0.3 mL/min for all the compounds.

Mass spectrometric measurements were carried out using a Waters Xevo TQ-S triple quadruple mass spectrometer coupled with an electrospray ionization (ESI) on a positive mode for all three compounds. Optimized MS-parameters were as follows: capillary 1.5 kV (terazosin and papaverine), 3.5 kV (levofloxacin), cone voltage 80 V (terazosin) and 8 V (terazosin D8), 22 V (papaverine and papaverine D3), 28 V (levofloxacin and levofloxacin D8), source temperature 150 °C, and desolvation temperature 500 °C (terazosin) or 450 °C (papaverine and levofloxacin). Nitrogen (AGA, Helsinki, Finland) was used as the desolvation gas (1000 L·h^−1^ terazosin, 800 L·h^−1^ papaverine and levofloxacin) and the cone gas (150 L·h^−1^), argon (AGA, Helsinki, Finland) as the collision gas. Multiple reaction monitoring (MRM) mode was employed for quantification. The mass transitions and their respective collision energies of studied compounds can be found in Table S3 of the Supplementary Materials. The resulting data were analyzed with Waters MassLynx V4.1 software. To consider the effect of melanin on the analyses, separate samples containing only melanin in PBS (with no test compound) were also studied.

### 2.7. Dissociation Constant Determination (K_d_, Sips Isotherm) 

The binding parameters were determined with Sips isotherm (Equation (2)) as described by Manzanares et al. [31].

B = (B_max_ × [L]^n^)/(K_d_^n^ + [L]^n^) (2)

where B is the amount of bound drug (nmol·mg^−^^1^); B_max_ is the maximum binding capacity (nmol·mg^−^^1^); [L] is the free drug concentration (µM); n is the heterogeneity index (assuming heterogeneity of the surface of the melanin particles); and K_d_ represents the dissociation constant (µM).

### 2.8. Prediction of Unbound Ligand Fraction In Vivo (Human RPE-Choroid) 

Unbound ligand fractions in vivo were calculated considering melanin binding as the only driver of nonspecific tissue binding. Cellular barriers and other nonspecific binding partners were not considered, as melanin binding has been shown to be the dominant factor in cellular kinetics for many compounds [16]. The predictions were made using binding parameters from the traditional assay (Sips isotherm) for the compounds where parameters were available (Table 2), assuming a total RPE-choroid compound concentration of 1 µM. For the other compounds (atropine, methotrexate, quinidine, penicillin G) the in vivo unbound fractions were calculated with Equation (3) [32] based on differences in melanin concentration in the in vitro assay and in vivo RPE-choroid, and similarly, the calculation assumed total tissue concentrations of approximately 0.3 µM (in vitro assay concentration for atropine, methotrexate, quinidine, and penicillin G). The unbound fractions for these four compounds were obtained from Pelkonen et al. [6], where the binding was studied at a melanin concentration of 3.6 mg/mL. As the binding of penicillin G and quinidine was studied only with the cassette method, where 34 compounds were incubated in the same assay, the binding of these compounds was corrected to correspond a single compound incubation with the binding of the two similarly high melanin binders tizanidine and ciprofloxacin. The unbound fractions of these two high binders were on average 2.1-fold higher in the cassette assay compared to the single compound incubation. Therefore, the unbound fractions of quinidine and penicillin G were divided by 2.1 to estimate the binding in a single compound assay.

f_unbound, in vivo_ (%) = (1/(D × (1/f_unbound in vitro_ − 1) + 1)) × 100 (3)

where D is the dilution factor, i.e., the ratio between in vivo and in vitro melanin concentration. To calculate the in vivo unbound ligand fractions, the melanin concentration in the human RPE-choroid was assumed to be 34.8 mg/mL as the measured average from blue and brown eyes (34.8 µg of melanin/mg tissue) in Menon et al. [33].

### 2.9. Statistical Analysis 

To evaluate the correlation of the melanin binding with traditional (Sips isotherm) and MST methods, the ratio of target capacity to K_d_ (MST) or K_d_^n^ (Sips isotherm) was determined. The correlation was determined with GraphPad Prism Software (version 7.04, San Diego, CA, USA) using nonparametric Spearman correlation. Target capacity for MST was melanin concentration in the assay (12.5 µM) and B_max_ multiplied with melanin concentration (0.5 mg/mL) in the case of Sips isotherm (each compound displaying different B_max_).

Linear regression was used to describe the relationship between the K_d_ values obtained with the MST and the predicted unbound ligand fractions in human RPE-choroid obtained with calculations described above. The linear regression was performed with GraphPad Prism Software (version 7.04, San Diego, CA, USA) by setting the intercept to x = y = 0.

## 3. Results

The MST trace curves and the target occupation (fraction bound) at each concentration of MST experiments are presented in the Supplementary Materials. Dissociation constants (K_d_) from MST and traditional melanin binding assays are presented in Table 2. Sips isotherm binding parameters of timolol, nadolol, propranolol, and chloroquine were obtained from previous studies [11,16] (Table 2). Binding parameters with either the traditional method or MST could not be determined for pazopanib due to its poor solubility, and K_d_ could not be determined for diclofenac, a drug with very weak melanin binding affinity [9,16]. With MST, the dissociation binding constants (K_d_) were successfully determined for 11/13 of the studied compounds (Table 2), and the values had a very wide range from 0.8 µM to more than 3400 µM.

**Figure 1:**
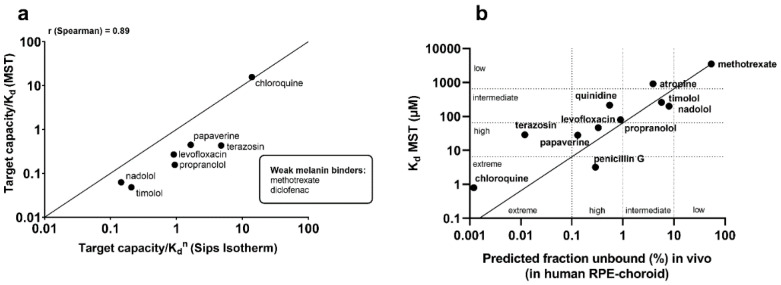
Melanin binding observed in MST experiments had good correlation between binding observed in traditional in vitro assays (**a**) and predicted in vivo binding (**b**). (**a**) Comparison of target capacity/K_d_ values from microscale thermophoresis (MST) and target capacity/K_d_^n^ from the traditional binding assay utilizing Sips isotherm resulted in a correlation of *r* = 0.89 (Spearman, *p* < 0.05; the solid line is the line of identity). (**b**) Linear regression (the solid line) between K_d_ values obtained with MST and predicted in vivo melanin binding enables crude predictions of in vivo melanin binding with the obtained equation of K_d_ (MST) = 65 * fraction unbound in vivo (%), the dotted lines indicate the classification limits into extreme (K_d_ < 6.5 µM), high (K_d_ 6.5–65 µM), intermediate (K_d_ 65–650 µM), and low (K_d_ > 650 µM) melanin binders (*y*-axis, B). The K_d_ limits were based on predicted fraction bound values in human RPE-choroid (fraction (%) bound of ligand; low (>10%), intermediate (1–10%), high (0.1–1%), and extreme (<0.1%).

The K_d_ values obtained with MST consider only one class of binding site, whereas the Sips isotherm (the traditional binding assay) depicts different binding energies and binding sites. Therefore, direct comparisons between these two parameters are not valid. As melanin has varying binding capacity for different ligands and different melanin concentrations can be used in the in vitro system, we calculated the target capacity/K_d_^n^ ratio for the traditional assay with the target capacities for the individual ligands (Table 2) and with the same melanin concentration as used in the MST assay (0.5 mg/mL). We evaluated the correlation between the obtained binding parameters considering the target capacity (Figure 1a). This approach is relevant to evaluate the melanin binding [13]. We observed a clear correlation (*r* = 0.89, Spearman, *p* < 0.05) between MST and the traditional assay. Furthermore, linear regression between the predicted unbound ligand fraction (%) in the human RPE-choroid and the K_d_ values obtained with MST (Figure 1b) resulted in the equation K_d_ (MST) = 65 × fraction unbound in vivo (%). This relationship was used to categorize compounds into four different binding classes, namely low, intermediate, high, and extreme melanin binders (Table 3) based on K_d_ (MST) and the corresponding predicted unbound fraction (%) in vivo. The predicted unbound ligand fractions in the human RPE-choroid and the categorization of each compound based on both MST and the traditional assay are presented in Table 4.

## 4. Discussion

Melanin binding of compounds can be measured with the label free MST method. The binding parameters show good correlation with the values from traditional experiments (*r* = 0.89, Figure 1a) that are based on the quantitation of the unbound drug in the incubation medium. Melanin binding of drugs is best described with the Sips isotherm that is based on non-specific surface binding at a range of different binding energies [30]. Binding experiments with the traditional approach allow data analysis using different binding isotherms (such as the Sips isotherm), whereas in the MST set-up we utilized the site-specific K_d_ analysis within the instrument’s software (which is the Langmuir isotherm, Equation (1)). Despite different detection principles (analysis of unbound ligand with LC–MS vs. analyzing the bound state of melanin with fluorescence detection in MST) and the different binding isotherms (Sips vs. Langmuir), good correlation and similar compound categorization were reached with the two methods (Figure 1a, Table 4). Interestingly, K_d_ can be determined with different modes even within the Monolith device—sometimes ligand-induced changes in the raw fluorescence counts are applicable instead of thermal-induced changes in the normalized fluorescence (thermophoresis). We saw ligand-induced changes in the initial fluorescence with several ligands in our study set and were able to determine similar K_d_ values with both MST and initial fluorescence modes (for additional information, see the Supplementary Materials).

The level of melanin binding is strongly dependent on the assay conditions (e.g., melanin and drug concentration in the assay [9,11]). For this reason, it is important to calculate the binding parameters (K_d_, B_max_, n). Due to the range of binding sites and energies on the melanin surface, the capacity of melanin binding varies among different compounds. Unlike in the case of well-defined binding sites (e.g., receptor binding), K_d_ values alone are not an adequate basis for melanin binding comparisons. Therefore, if one is interested in comparing MST and Sips isotherm, other parameters such as B_max_ and n, describing the heterogeneity, should be taken together with binding affinity (K_d_) and target binding capacity (Figure 1a).

As the most common melanin type in the posterior eye [34] and in melanoma cells [35] is eumelanin, the synthetic dopa-melanin was a relevant choice as the melanin source for this study. We performed the binding experiments with water-soluble melanin nanoparticles to avoid melanin adsorption to the capillary walls and polymer aggregation during the thermophoresis experiment. This was necessary, because melanin is hygroscopic and poorly soluble in water, and it adheres to various plastics and glassware materials. In their original work on melanin nanoparticle preparation, Fan et al. [30] hypothesized that nanoparticles display decreased interchain π–π aggregation of conjugated polymer units, and hydrophilic hydroxy groups are exposed to the particle surface making the nanoparticles water-soluble. Based on our results, it seems that possible surface changes of melanin are not causing significant changes on melanin binding of drugs. Overall, the preparation of melanin nanoparticles was crucial for the use of MST, because aggregation and adsorption were seen with unmodified synthetic and isolated melanin (porcine RPE [9]; data not shown). Furthermore, the previous work of Jakubiak et al. [36] showed correlation among the drug binding to different melanin types (porcine RPE-choroid, sepia, and synthetic), supporting the notion that melanin binding is a robust phenomenon over melanin from different sources.

We demonstrate here that MST yields similar binding results with traditional binding assays that often utilize LC–MS/MS analytics. Compared to the traditional approach, the MST method is more cost-effective. The price of MST instrumentation (≈€100,000) is several times less than the price of LC–MS/MS (≈€300,000). The benefits of the MST approach include also the short duration of the analyses and its methodological simplicity. Importantly, melanin binding can be performed label-free (without fluorescence labeling), and development of chemical drug quantitation methods is avoided completely. The MST measurement of three replicate samples takes less than an hour, and the built-in software enables immediate and partly automated determination of K_d_ values after the experiment. The MST assays can be performed at low quantities of ligands and melanin, even at an incubation volume of 20 µL. Strong auto-fluorescence of the ligand may interfere with the assay, and in these cases, traditional determination of unbound drug concentration may be needed. For many compounds, the concentration range and setting of our study can be directly used to determine melanin binding. On occasion, extremely low water-solubility of the drug may hinder reliable determination of K_d_ values. For example, poor water-solubility of pazopanib limited the concentration range to the values below 4 µM. Therefore, fitting of the data resulted in an extremely wide confidence range (1100 µM), even though the categorization correctly classified pazopanib as an extreme melanin binder (K_d_ ≈ 7.8 µM, Supplementary Materials). Pazopanib displays a high affinity towards melanin [27] and long retention in pigmented ocular tissues [18]. Overall, MST serves as a promising tool for categorizing melanin binders, as long as the studied compounds do not comprise auto-fluorescence properties or poor water-solubility in the studied range.

Melanin binding is a relevant factor in some fields of pharmacokinetics, for example drug distribution to the pigmented cells in the eye, melanoma tissue, and substantia nigra. Melanin binders may generate melanosomal drug depots for sustained drug retention and release in the cells and tissues. This is a known phenomenon and an attractive option for prolonging intravitreal drug dosing intervals and maintenance of therapeutic drug levels in the posterior eye tissues (choroid, RPE) [17]. High melanin binding may even enable non-invasive (e.g., oral administration, eye drops) drug delivery to the pigmented tissues in the posterior eye segment. Pharmacokinetic classification of different melanin binders can be utilized as an additional tool in drug discovery. However, special attention should be pointed to the relationship between the in vitro binding categorization and the predicted unbound fraction in human RPE-choroid (Table 3). Melanin concentration in the RPE-choroid is significantly higher in vivo compared to concentrations used in the in vitro systems (~35 mg/mL in vivo [37] compared to 0.5–5 mg/mL in vitro used generally in the field). Therefore, the compounds displaying low melanin binding in vitro (K_d_ > 650 µM) can be up to 90% bound to melanin in the human RPE-choroid, and therefore, even compounds with low melanin affinity in vitro might display pharmacokinetic and pharmacodynamic differences in subjects with differences in the pigmentation. For instance, the pharmacokinetics of low and intermediate melanin binders is affected by the pigmentation in the iris and ciliary body. Atropine and pilocarpine do not have high affinity towards melanin in vitro [9], but their pharmacokinetics and ocular responses are affected by the iris pigmentation [13,14,15]. However, recent findings suggest that the high in vitro melanin binders display long retention in the pigmented ocular tissues—intermediate binders may stay in the pigmented tissues for days, whereas high and extreme binders may show retention for weeks or months [17]. Therefore, drug discovery programs benefit from tools that effectively distinguish high and extreme melanin binders.

As the free concentration determines the compound’s pharmacological response, the potency of the compound is an important criterion. Therefore, potent compounds may have extended and effective concentrations of free drug, even if most of the drug in the cells would be pigment bound [8]. In the case of low potency drug compounds, melanin binding may easily lower the free drug concentrations below the threshold level, resulting in the loss of therapeutic levels [38]. Therefore, screening of melanin binding should be linked to information on drug potency and, for selected cases, assays on melanosomal drug uptake in the cells [39], unbound cellular partition coefficients (Kp_uu_) [16], and in vivo experiments [11]. The MST method is useful in the early phase to select the compounds for more time-consuming and expensive experiments. The MST screening and categorization method for melanin binders can be used as a selection tool in drug discovery programs to find compounds that target melanin containing tissues. On the other hand, it can also give early indications about unintentional accumulation of drug candidates to the pigmented tissues.

## 5. Conclusions

In conclusion, MST is an efficient tool for screening and categorization of compounds in terms of melanin binding. The method is target-based, and in most cases, binding of ligands can be determined with the same procedural rapidly with a small quantity of compound. In addition to ranking compounds with their K_d_ values, the method can be used to predict the unbound ligand fractions in human RPE-choroid in vivo. The method is valuable in ocular drug discovery but can be applied also in other fields in which melanin binding of drugs is relevant.

## Figures and Tables

**Table 1:** Studied compounds and their concentrations in the MST and traditional melanin binding assays.

Compound	LogD_7.4_	pK_a_ (MB)	pK_a_ (MA)	Vendor (Cat n:o)	Concentration Range in the MST Binding Assay (µM)	Concentration Range in the Traditional Binding Assay (µM)
Atropine	−0.41	15.15	9.39	Fluka (11330)	0.005–500	n.d.
Chloroquine	1.43	-	10.32	Sigma Aldrich (C6628)	0.005–100	0.25–500 ^1^
Ciprofloxacin	−0.87	5.56	8.77	BioChemica (17850)	0.005–500	n.d.
Diclofenac	1.10	4	-	Sigma Aldrich (D6899)	0.005–500	0.5–500 ^1^
Levofloxacin	−0.51	5.35	6.72	Sigma Aldrich (28266)	0.005–500	0.25–500
Methotrexate	−6.56	3.25	2.81	Fluka (PHR1396)	0.005–500	0.25–250 ^2^
Nadolol	−1.44	13.59	9.76	Sigma Aldrich (N-1892)	0.005–500	0.5–500 ^2^
Papaverine	3.06	-	6.03	Sigma Aldrich (P5310)	0.005–250	0.25–250
Pazopanib	3.55	10.41	4.36	Sigma Aldrich (CDS023580)	0.25–4	n.d.
Propranolol	0.36	14.09	9.67	Sigma Aldrich (P-0884)	0.005–500	0.5–500 ^1^
Penicillin g	−2.29	3.53	-	Sigma Aldrich (P-7794)	0.005–500	n.d.
Terazosin	0.95	-	7.24	Sigma Aldrich (T4680)	0.005–250	0.25–500
Quinidine	0.86	13.89	9.05	Sigma Aldrich (22600)	0.005–500	n.d.
Timolol	−0.97	14.08	9.76	Sigma-Aldrich (T6394-250MG)	0.005–500	0.25–250 ^2^

^1^ Rimpelä et al. [16]; ^2^ Rimpelä et al. [11]; n.d., not studied; MA, the most acidic; MB, the most basic. LogD_7.4_ and pKa values from references [11,16] or generated with Chemicalize (ChemAxon, Budapest, Hungary).

**Table 2:** Melanin binding parameters determined with microscale thermophoresis (MST) and traditional binding assay (Sips isotherm). Target capacity for Sips isotherm translated to MST assay using 0.5 mg/mL melanin concentration.

Compound	MST		Traditional Method (Sips Isotherm) ^1^			
	K_d_ ± K_d_ Confidence (µM)	Target Capacity (µM)	K_d_ ± SE ^2^ (µM)	B_max_ ± SE ^2^ (nmol/mg)	n ± SE ^2^	Target Capacity ± SE ^2^ (µM)
Terazosin *	29 ± 28	12.5	46 ± 16	112 ± 13	0.64 ± 0.018	56 ± 6.5
Penicillin G	3.2 ± 7.6	12.5	not studied	not studied	not studied	not studied
Chloroquine	0.8 ± 7.7	12.5	76 ± 23	380 ± 40	0.605 ± 0.012	190 ± 20
Levofloxacin	46.5 ± 40.1	12.5	157 ± 56	72 ± 11	0.73 ± 0.02	36 ± 5.5
Papaverine *	28 ± 23	12.5	66 ± 18	66 ± 8	0.715 ± 0.015	33 ± 4
Propranolol *	80 ± 92	12.5	163 ± 100	176 ± 60	0.89 ± 0.05	88 ± 30
Nadolol *	200 ± 33	12.5	340 ± 200	68 ± 24	0.94 ± 0.05	34 ± 12
Timolol	260 ± 128	12.5	120 ± 30	39 ± 6	0.95 ± 0.03	19.5 ± 3
Quinidine *	214 ± 29	12.5	not studied	not studied	not studied	not studied
Atropine	912 ± 1318	12.5	not studied	not studied	not studied	not studied
Methotrexate *	3500 ± 13,000	12.5	no binding ^1^	no binding ^1^	no binding ^1^	no binding ^1^
Diclofenac	K_d_ could not be determined	12.5	no binding ^1^	no binding ^1^	no binding ^1^	no binding ^1^

The K_d_ values obtained with the initial fluorescence mode were utilized for compounds marked with *. ^1^ Sips isotherm binding parameters of timolol, nadolol, propranolol, and chloroquine were obtained from previous studies [11,16]; methotrexate and diclofenac did not display melanin binding [16]. ^2^ Sips isotherm parameter values (±standard error, SE).

**Table 3:** Limits for the binding classes based on the linear correlation seen in Figure 1b.

Class	Fraction Unbound in Vivo (%)	K_d_ (MST)
Low	>10%	>650 µM
Intermediate	1–10%	65–650 µM
High	0.1–1%	6.5–65 µM
Extreme	<0.1%	<6.5 µM

**Table 4:** Classification of compounds based on the traditional assay and MST results, according to the classes in Table 3. The calculated in vitro and in vivo fractions unbound (%) are presented as a reference.

Compound	Based on Traditional Assay	Based on K_d_ from MST	Fraction Unbound in Vitro (%) (1 mg/mL Melanin)	Fraction Unbound in Vivo * (%)	Calculation Method
Chloroquine	extreme	extreme	0.41	0.0012	Sips binding parameters ^a^
Terazosin	extreme	high	2.9	0.012	
Levofloxacin	high	high	28	0.33	
Propranolol	high	intermediate	32	0.9	
Papaverine	high	high	15	0.13	
Nadolol	intermediate	intermediate	78	8.0	
Timolol	intermediate	intermediate	71	5.7	
Atropine	intermediate	low	59	3.9	Dilution equation (based on binding data from [6]) ^b^
Methotrexate	low	low	98	54	
Quinidine	high	intermediate	16	0.55	Dilution equation (based on binding data from [6]) ^b^
Penicillin G	high	extreme	9.2	0.29	

* based on the traditional assay. ^a^ Where binding parameters were available for Sips isotherm, calculations were made for 1 µM ligand concentration. ^b^ Calculations are made based on studied concentration in the reference [9] (~0.3 µM).

## Supplementary Materials

The following are available online at https://www.mdpi.com/1999-4923/12/6/554/s1: Figure S1. Microscale thermophoresis trace curves showing normalized fluorescence during the experiment and the time region which was used to determine the K_d_, Table S1. The hot regions in the MST curves used to determine the K_d_ values, Table S2. K_d_ values determined with MST (F_norm_) or initial fluorescence modes, Figure S2. MST was able to distinguish compounds with different binding affinities towards melanin. Table S3. Mass spectrometric conditions used in the compound quantification.
