# Supplementary material for: Microscale Thermophoresis as a Screening Tool to Predict Melanin Binding of Drugs
Source: Pharmaceutics. 2020 Jun 16;12(6):554. doi: 10.3390/pharmaceutics12060554 (PMC7355663; doi:10.3390/pharmaceutics12060554)
Supplement: Supplementary file 1 [file pharmaceutics-12-00554-s001.pdf]

*pharmaceutics*

# Supplementary Materials: Microscale Thermophoresis as a Screening Tool to Predict Melanin Binding of Drugs

**Laura Hellinen, Sina Bahrpeyma, Anna-Kaisa Rimpelä, Marja Hagström, Mika Reinisalo and Arto Urtti**

**The (microscale thermophoresis) MST traces** are presented in the Supplementary Figure S1 below. The time regions from which the changes in the $F_{norm}$ (and consequently binding affinity) were determined are displayed in the Table S1. The cold region was from -1.00 to 0 seconds (i.e. the cold region started one second before infrared laser was switched on to which the cold region ends).

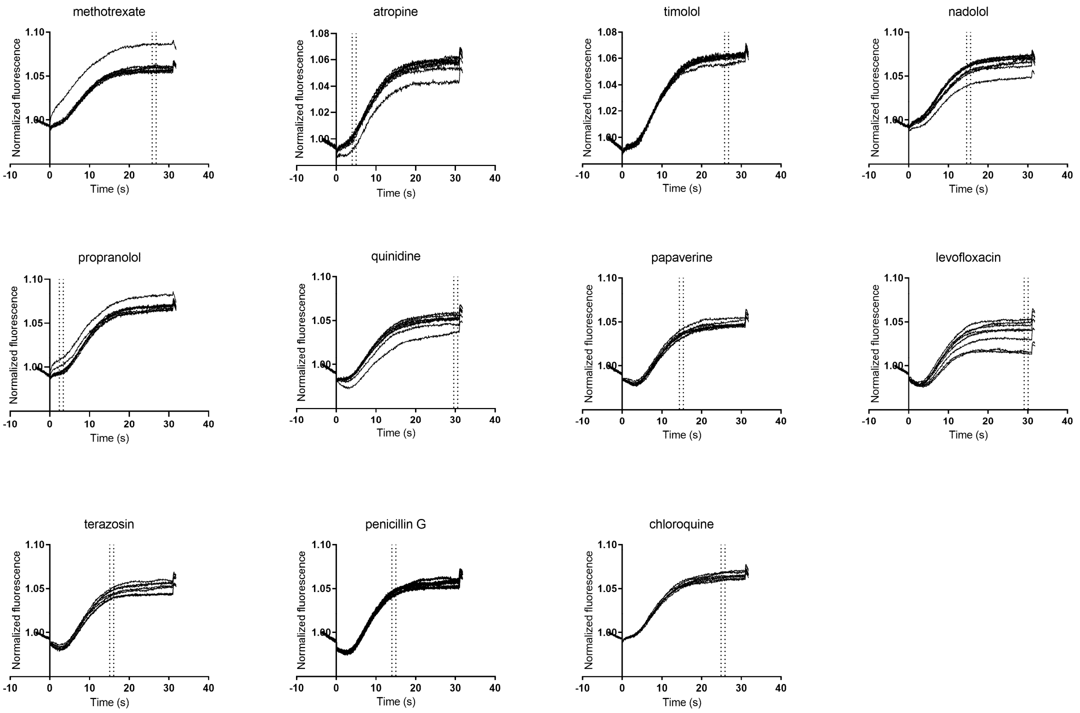


**Figure S1.** Microscale thermophoresis trace curves showing normalized fluorescence during the experiment and the time region which was used to determine the $K_d$.

***Table S1.*** The hot regions in the MST curves used to determine the $K_d$ values.

| Compound | MST hot region (s) used to determine $F_{norm}$ |
|---|---|
| **timolol** | 25.8-26.8 |
| **nadolol** | 14.6-15.6 |
| **atropine** | 4.0-5.0 |
| **quinidine** | 29.6-30.6 |
| **chloroquine** | 24.9-25.9 |
| **penicillin G** | 14.0-15.0 |
| **terazosin** | 15.1-16.1 |
| **papaverin** | 14.4-15.4 |
| **levofloxacin** | 29.1-30.1 |

**$K_d$ determination strategies.** The $K_d$ values were tested in both initial fluorescence and the above described MST modes within the MO Affinity Analysis Software (Fig. S1, Table S1) and the $K_d$ values obtained with each of these modes are described in the Table S2 below. Since the user manual incorporated into the software (Nanopedia) advices to utilize the initial fluorescence mode to determine $K_d$ if ligand-induced fluorescence changes are observed, the initial fluorescence mode was used whenever fitting with raw fluorescence counts was successful. The fitting of the two modes are the same, but in the case of initial fluorescence mode, the raw fluorescence counts measured before the heating were used instead of normalized fluorescence values ($F_{norm}$). In the case of levofloxacin, the $K_d$ fitting with initial fluorescence was unsuccessful with 60 % excitation, even though trend towards ligand-induced fluorescence change could be visually seen. The fitting with initial fluorescence was successful once higher excitation power (80 %) was used and the $K_d$ is within the same range than the value obtained with the MST mode using 60 % excitation.

***Table S2.*** $K_d$ values determined with MST ($F_{norm}$) or initial fluorescence modes.

| Compound | MST mode | | Initial fluorescence mode | |
|---|---|---|---|---|
| | **$K_d$ (μM)** | **$K_d$ confidence[1] (μM)** | **$K_d$ (μM)** | **$K_d$ confidence[1] (μM)** |
| **atropine** | >900 | 1300 | Failed | - |
| **diclofenac** | Failed | - | Failed | - |
| **methotrexate*** | >10 000 | 200 000 | 3 500 | 13 000 |
| **timolol** | 260 | 130 | Failed | - |
| **nadolol*** | 260 | 52 | 200 | 33 |
| **propranolol*** | 230 | 124 | 80 | 92 |
| **quinidine*** | 370 | 370 | 214 | 29 |
| **papaverine*** | 93 | 150 | 28 | 23 |
| **pazopanib*** | Failed | - | 7.8 | 1100 |
| **levofloxacin** | 47 | 40 | [2]Failed/54 | [2]Failed/10 |
| **chloroquine** | 0.8 | 7.7 | Failed | - |
| **penicillin G** | 3.2 | 7.6 | Failed | - |
| **terazosin*** | 96 | 193 | 29 | 28 |

The $K_d$ values obtained with the initial fluorescence mode were utilized for compounds marked with*
[1]$K_d$ confidences were obtained within the MO Affinity Analysis Software. With the confidence of 68 %, the $K_d$ is in the reported range. [2]Unsuccessful with 60 % excitation power, reported value gained with 80 % excitation.

**The target (melanin) occupation** (fraction (%) bound values) in each of the studied ligand concentration are presented in the Figure S2 below. The categorization of the compounds into low ($K_d$ >650 μM) intermediate ($K_d$ 65-650 μM), high ($K_d$ 6.5-65 μM) and extreme ($K_d$ < 6.5 μM) melanin-binders were based on the predicted ligand binding in human RPE-choroid in vivo (fraction (%) bound of ligand; low (>10%), intermediate (1-10 %), high (0.1-1 %) and extreme (< 0.1 %).

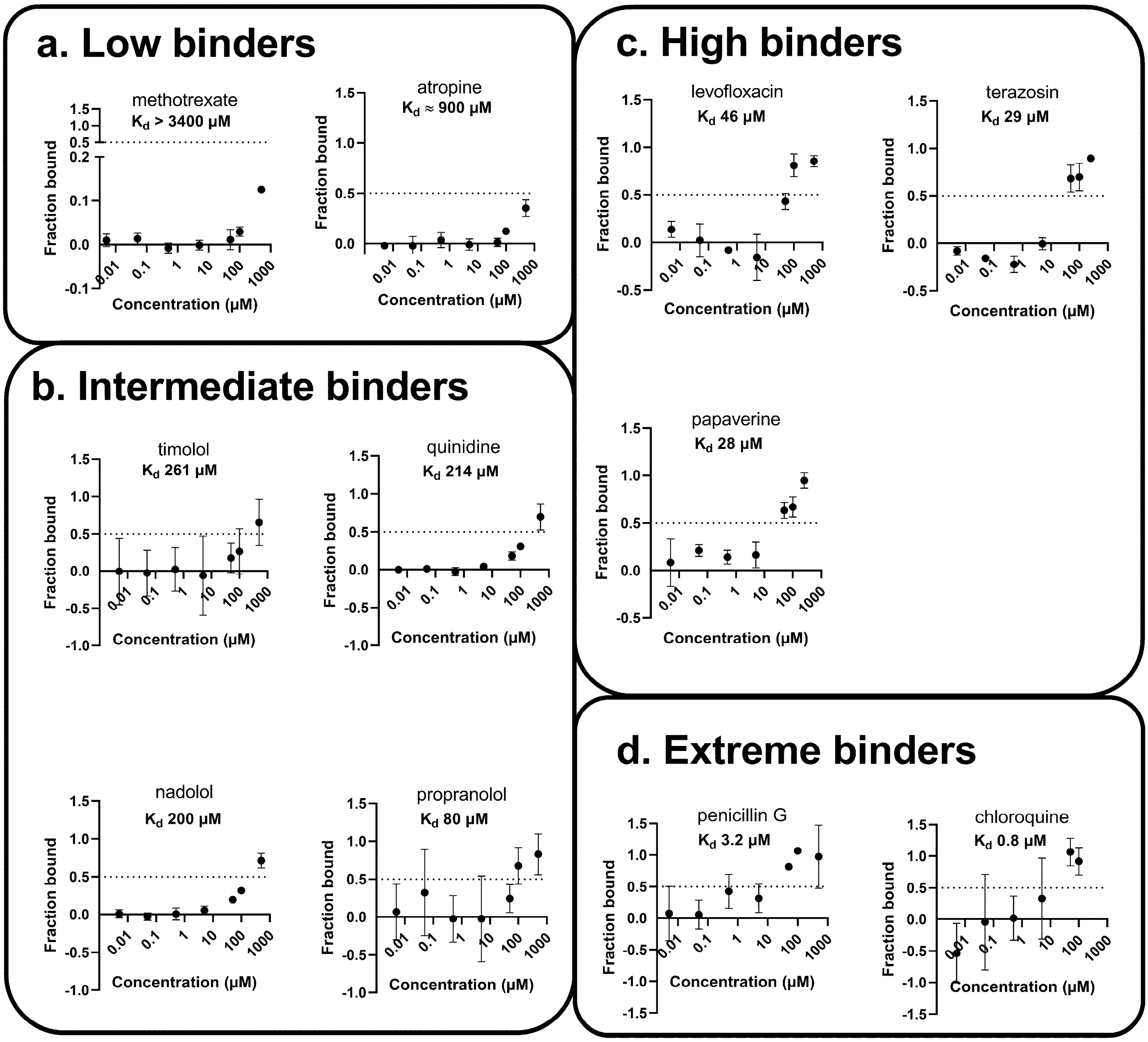


**Figure S2. MST was able to distinguish compounds with different binding affinities towards melanin.** Fraction of melanin in bound state at studied range of concentration according to MST analysis for (A) low binders, (B) intermediate melanin binders, (C) high binders, and (D) extreme binders.

***Table S3.*** Mass spectrometric conditions used in the compound quantification.

| Compound | Precursor m/z | Fragment m/z | Collision Energy V | Ionization |
|---|---|---|---|---|
| **terazosin** | 388.27 | 290.20 | 24 | ESI+ |
| **terazosin** | 388.27 | 247.14 | 32 | ESI+ |
| **terazosin D8** | 396.26 | 298.25 | 28 | ESI+ |
| **terazosin D8** | 396.26 | 251.80 | 34 | ESI+ |
| **papaverine** | 340.18 | 202.08 | 26 | ESI+ |
| **papaverine** | 340.18 | 171.08 | 38 | ESI+ |
| **papaverine D3** | 343.24 | 205.14 | 26 | ESI+ |
| **papaverine D3** | 343.24 | 174.13 | 42 | ESI+ |
| **levofloxacin** | 362.11 | 318.21 | 18 | ESI+ |
| **levofloxacin** | 362.11 | 261.14 | 26 | ESI+ |
| **levofloxacin D8** | 370.24 | 326.33 | 20 | ESI+ |
| **levofloxacin D8** | 370.24 | 265.28 | 28 | ESI+ |
